# Delayed Diagnosis and Evolving Trends in Gastric Cancer During and After COVID-19: A Comparative Study of Staging, *Helicobacter pylori* Infection and Bleeding Risk in Western Romania

**DOI:** 10.3390/diagnostics15080950

**Published:** 2025-04-09

**Authors:** Patricia Serena, Bogdan Miutescu, Eyad Gadour, Calin Burciu, Ruxandra Mare, Renata Bende, Edward Seclăman, Giovanni Aragona, Luca Serena, Roxana Sirli

**Affiliations:** 1 Division of Gastroenterology and Hepatology, Department of Internal Medicine II, “Victor Babes” University of Medicine and Pharmacy Timisoara, Eftimie Murgu Square 2, 300041 Timisoara, Romania; patricia.lupulescu@umft.ro (P.S.); mare.ruxandra@umft.ro (R.M.); bende.renata@umft.ro (R.B.); sirli.roxana@umft.ro (R.S.); 2 Advanced Regional Research Center in Gastroenterology and Hepatology, “Victor Babes” University of Medicine and Pharmacy Timisoara, 300041 Timisoara, Romania; calin.burciu@umft.ro; 3 Multi-Organ Transplant Centre of Excellence, Liver Transplantation Unit, King Fahad Specialist Hospital, Dammam 32253, Saudi Arabia; eyadgadour@doctors.org.uk; 4 Department of Medicine, Faculty of Medicine, Zamzam University College, Khartoum 11113, Sudan; 5 Department of Gastroenterology, Faculty of Medicine, Pharmacy and Dental Medicine, “Vasile Goldis” West University of Arad, 310414 Arad, Romania; 6 Department IV—Biochemistry and Pharmacology, Faculty of Medicine, “Victor Babes” University of Medicine and Pharmacy Timisoara, 2nd Eftimie Murgu Square, 300041 Timisoara, Romania; eseclaman@umft.ro; 7 Gastroenterology and Hepatology Unit, Guglielmo da Saliceto Hospital, 29121 Piacenza, Italy; g.aragona@ausl.pc.it; 8 Anaesthesia and Intensive Care Department, Guglielmo da Saliceto Hospital, 29121 Piacenza, Italy; dr.serenaluca@gmail.com

**Keywords:** gastric cancer, oncology, *Helicobacter pylori*

## Abstract

**Background and Objectives:** Gastric cancer (GC) remains a leading cause of cancer mortality worldwide, and the COVID-19 pandemic posed new barriers in diagnosis and management. This study aimed to assess whether pandemic-related healthcare disruptions resulted in more advanced GC stages at presentation. We additionally examined the role of *Helicobacter pylori* (*H. pylori*) across non-cardia GC (NCGC) versus cardia GC (CGC) and evaluated the risk factors of upper gastrointestinal (GI) bleeding. **Methods:** A retrospective cohort of 121 adult patients with GC was enrolled from a tertiary Gastroenterology Unit in Western Romania, spanning pre-pandemic (March 2018–February 2020), pandemic (March 2020–February 2022), and post-pandemic (March 2022–February 2024) periods. Demographic profiles, TNM staging, histopathology, *H. pylori* status, and clinical outcomes—including GI bleeding—were extracted from medical records. **Results:** An increase in advanced GC (Stage III–IVB) was noted in the post-pandemic period (69.4% vs. 53.3% pre-pandemic; *p* = 0.021). *H. pylori* positivity remained higher in NCGC (70.6%) compared to CGC (44.6%; overall *p* = 0.041); however, CGC cases showed a rise in *H. pylori* prevalence post-pandemic (36.4% to 55.6%). One-year mortality was driven by an advanced stage (hazard ratio [HR] = 2.74, *p* = 0.002), diagnosis during the COVID-19 pandemic (HR = 1.66, *p* = 0.010), and age ≥70 years (HR = 1.88, *p* = 0.043). **Conclusions:** Our findings demonstrate that delayed diagnostic endoscopy correlated with a higher proportion of advanced GC in the post-pandemic phase. *H. pylori* was strongly linked to NCGC, though CGC showed an increasing trend in *H. pylori* prevalence. Patients on antithrombotic agents faced increased GI bleeding risks.

## 1. Introduction

Gastric cancer (GC) is a major global health concern, ranking among the fifth most common malignancies and the fourth leading cause of cancer-related mortality worldwide [1,2]. According to 2020 estimates, GC accounted for roughly 1.1 million new cases and remains associated with significant morbidity and mortality [3]. Risk factors for GC include chronic *Helicobacter pylori* (*H. pylori*) infection, dietary components high in salt and nitrites, and tobacco use [4,5,6]. The World Health Organization has classified *H. pylori* as a Class I carcinogen, and epidemiological studies consistently link this pathogen to non-cardia gastric cancer (NCGC), with a more variable association in cardia gastric cancer (CGC) [7,8,9,10,11,12,13,14,15,16].

The COVID-19 pandemic introduced unprecedented challenges to healthcare. Many centers postponed or limited non-emergency endoscopies, which potentially delayed GC diagnoses [17,18,19]. In Romania, organizational changes and patient avoidance of hospitals out of fear of infection further decreased endoscopic procedures and early detection of GC. Late presentation is highly detrimental, as advanced-stage disease significantly diminishes survival [16].

Upper gastrointestinal (GI) bleeding is a life-threatening complication in GC, occurring in 10–58% of patients depending on tumor location and extent [9,20]. Antiplatelet or anticoagulant therapies, while essential for cardiovascular protection, may exacerbate bleeding in the presence of a tumor [21,22,23,24]. Balancing these therapies against bleeding risk is an ongoing clinical dilemma.

The nature of gastric cancer care is multifaceted—where staging, Helicobacter pylori status, and bleeding risk frequently intersect in clinical practice. Therefore, we aimed (1) to evaluate changes in GC staging before, during, and after the COVID-19 pandemic, (2) to analyze *H. pylori* prevalence in CGC versus NCGC, and (3) to assess the impact of antiplatelet/anticoagulant use on upper GI bleeding risk. By comparing three time periods (pre-pandemic, pandemic, and post-pandemic), we offer insights into how healthcare disruptions affected GC diagnosis and outcomes.

## 2. Materials and Methods

### 2.1. Study Design and Setting

The PICO statement of this study was considered as follows: Population (P): adult patients (*n* = 121) with confirmed gastric cancer in a tertiary Gastroenterology Unit in Western Romania. Intervention (I): comparison of gastric cancer diagnosis and care before, during, and after the COVID-19 pandemic. Comparison (C): three time periods (pre-pandemic, pandemic, and post-pandemic) reflecting variations in healthcare access. Outcome (O): tumor stage at presentation, Helicobacter pylori infection rates in cardia and non-cardia gastric cancers, risk of upper GI bleeding, and one-year mortality.

This retrospective cohort study was conducted at a tertiary Gastroenterology Unit in Western Romania, focusing on adult patients (≥18 years) diagnosed with gastric cancer over a six-year interval (March 2018–February 2024). We divided this timeframe into three distinct periods to capture any impact of the COVID-19 pandemic: pre-pandemic (March 2018–February 2020), pandemic (March 2020–February 2022), and post-pandemic (March 2022–February 2024). As a tertiary referral center, our facility receives patients from smaller regional hospitals, expanding the representativeness of the study population.

All patients underwent esophagogastroduodenoscopy (EGD) for evaluation of upper GI symptoms (including alarm features such as anemia, weight loss, and dysphagia) or acute bleeding episodes. EGDs were performed using Olympus Evis EXERA III (CV-190) and GIF-HQ190 endoscopes (Olympus corp., Tokyo, Japan). During the endoscopic procedures, the patients were sedated with benzodiazepines (Midazolam), fentanyl, and propofol, and at least four biopsies were taken from the tumor site for histopathological confirmation of GC. *H. pylori* status was assessed via histology only. Tumor staging was completed using the TNM classification system (8th edition) [1,2], confirmed by computed tomography (CT) of the abdomen and pelvis to evaluate local extension and distant metastases.

The latest TNM classification for gastric cancer, as outlined by the American Joint Committee on Cancer (AJCC), organizes the disease based on three key factors: the extent of the primary tumor (T), the absence or presence of regional lymph node involvement (N), and the absence or presence of distant metastasis (M). The primary tumor (T) is categorized from T1, indicating tumor invasion into the lamina propria or submucosa, to T4a and T4b, where T4a involves tumor penetration through the serosa without invasion into adjacent structures and T4b indicates invasion into nearby structures. Lymph node involvement (N) is classified from N0, with no regional lymph node metastasis, to N3, which is further subdivided into N3a (7–15 metastatic nodes) and N3b (more than 15). Metastasis (M) is categorized as M0, indicating no distant metastasis, or M1, where there is evidence of distant metastasis.

### 2.2. Patient Selection, Inclusion/Exclusion Criteria, and Definitions

The inclusion criteria were (1) histologically confirmed gastric adenocarcinoma or other malignant GC subtypes (e.g., lymphoma, undifferentiated carcinoma); (2) availability of complete medical records detailing demographic variables, comorbidities, presenting symptoms, *H. pylori* status, staging, and treatments; and (3) admission dates falling within the designated study intervals. Patients were categorized as having cardia gastric cancer (CGC) if the tumor primarily involved the gastroesophageal junction/cardia region, whereas non-cardia gastric cancer (NCGC) encompassed lesions arising from the body, antrum, or pylorus.

The exclusion criteria included (1) histologically negative or inconclusive biopsy results; (2) incomplete clinical data (e.g., missing endoscopic or imaging reports); (3) concurrent malignant conditions of other GI sites (e.g., esophageal cancer) that could confound staging and outcomes; and (4) patients lost to follow-up within 14 days of diagnosis, preventing reliable staging or outcome assessments.

Upper GI bleeding was defined as the presence of hematemesis, melena, or endoscopic evidence of active bleeding. “Advanced stage” referred to TNM Stage III or IV, while “early stage” comprised Stages I or II. Anemia was defined by a hemoglobin level < 12 g/dL in women or <13 g/dL in men. Antiplatelet/anticoagulant use included aspirin, clopidogrel, vitamin K antagonists (VKA), and direct oral anticoagulants (DOACs) initiated prior to or during admission.

### 2.3. Data Collection Procedures and Variables

Data were extracted from both paper-based and electronic health records by two independent reviewers trained in epidemiologic methods. Demographic variables included age, gender, and body mass index (BMI). Clinical variables encompassed presenting symptoms (weight loss, dysphagia, vomiting, early satiety, weakness), vital signs, and relevant laboratory parameters (hemoglobin, platelets, international normalized ratio [INR]). We also captured comorbidities such as hypertension, diabetes, and heart failure, as well as habits like smoking and alcohol consumption.

Histopathological categories were grouped as adenocarcinoma (including tubular, papillary, mucinous, mixed subtypes), signet-ring (poorly cohesive) cell carcinoma, lymphoma (diffuse large B-cell or follicular), and undifferentiated carcinoma. Tumor localization was designated as either CGC or NCGC based on endoscopic and radiologic findings. *H. pylori* status was determined via histological staining of biopsy specimens. Upper GI bleeding was recorded if documented in the endoscopy or clinical notes. Antiplatelet and anticoagulant therapies were noted at admission, with additional details about dosage and indication where available.

### 2.4. Statistical Analysis

A priori power analysis was conducted using G*Power software (version 3.1, Heinrich Heine University, Düsseldorf, Germany) to estimate the required sample size for detecting a clinically meaningful shift in the proportion of advanced-stage (Stage III–IV) gastric cancer cases across the three study periods. Based on prior regional data suggesting a 20% increase in advanced-stage disease post-pandemic, a power of 80%, and a two-sided alpha of 0.05, the calculation yielded a minimum total sample size of approximately 110 participants. Ultimately, 121 patients met the inclusion criteria, exceeding this threshold and thus providing adequate power to detect statistically significant differences in tumor stage and associated variables.

All statistical analyses were performed using IBM SPSS Statistics (version 27) and Stata/SE (version 18). Descriptive statistics were presented as means (±standard deviations) or medians (IQR) for continuous variables and counts (percentages) for categorical variables. When comparing three groups (pre-pandemic, pandemic, post-pandemic), we used one-way ANOVA for normally distributed continuous variables or the Kruskal–Wallis test if distributions were skewed. For categorical comparisons, we used chi-square or Fisher’s exact tests, as appropriate, with a significance threshold of *p* < 0.05. For each row in the multi-row tables, a separate *p*-value was calculated to examine differences among the three time periods.

We conducted logistic regression to evaluate factors associated with upper GI bleeding, incorporating significant variables from univariate analyses (*p* < 0.10) into a multivariable model. Cox proportional hazards models assessed one-year mortality, reporting hazard ratios (HRs) with 95% confidence intervals (CIs). The variables tested included age, advanced stage, *H. pylori* infection, tumor location, and antithrombotic therapy. The proportional hazards assumption was verified graphically and via goodness-of-fit tests. Statistical significance was determined at *p* < 0.05 for all analyses, and all tests were two-sided.

## 3. Results

**Figure 1:**
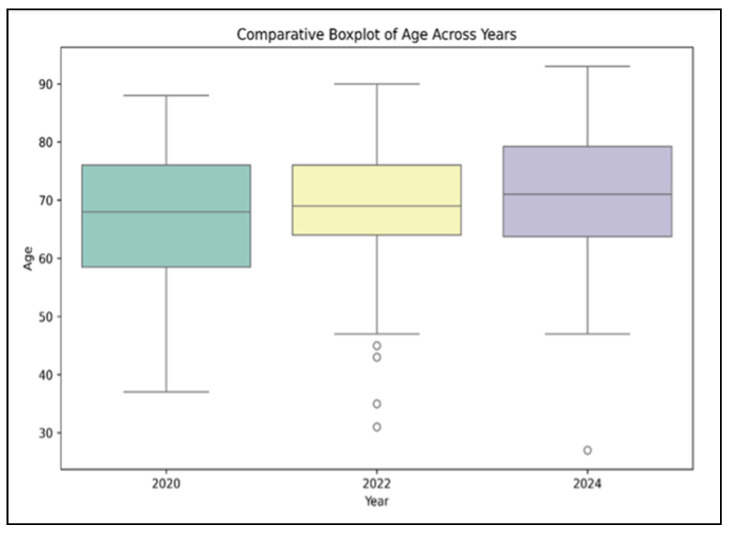
Comparative boxplot of age distributions across three years: 2020, 2022, and 2024.

Regarding patient demographics, the mean age of patients increased slightly across the three cohorts, from 66.2 years (±9.7) in the pre-pandemic group (*n* = 45) to 68.5 years (±10.3) during the pandemic (*n* = 40) and 70.4 years (±11.2) in the post-pandemic period (*n* = 36), although this incremental rise did not reach statistical significance (*p* = 0.149), as presented in Figure 1. Male sex predominated in all three groups (64.4% pre-pandemic; 70.0% pandemic; 75.0% post-pandemic), but again, no statistically significant difference was observed (*p* = 0.417).

In terms of comorbid conditions, hypertension was present in roughly half to two-thirds of participants (62.2% pre-pandemic; 55.0% pandemic; 69.4% post-pandemic), and heart failure was documented in 40–44% of individuals across the three groups. Diabetes rates remained relatively similar (20.0% pre-pandemic; 22.5% pandemic; 19.4% post-pandemic). Anemia affected more than three-quarters of patients in each cohort, with percentages ranging from 80.6% to 85.0%. Obesity (BMI > 30) and smoking also showed consistent prevalence with no significant differences: obesity hovered around 30–33%, and smoking varied between 38 and 45%. Overall, the distribution of comorbidities and lifestyle factors did not vary significantly across the three timeframes, as indicated by *p*-values exceeding 0.05 (Table 1).

In the pre-pandemic cohort (*n* = 45), the distribution by stage was as follows: Stage I in 5 patients (11.1%), Stage IIA in 8 (17.8%), Stage IIB in 6 (13.3%), Stage III in 9 (20.0%), Stage IVA in 13 (28.9%), and Stage IVB in 4 (8.9%), with a *p*-value of 0.679 for Stage I comparisons. During the pandemic period (*n* = 40), the numbers were Stage I in 3 patients (7.5%), Stage IIA in 7 (17.5%), Stage IIB in 5 (12.5%), Stage III in 10 (25.0%), Stage IVA in 5 (12.5%), and Stage IVB in 10 (25.0%), with corresponding *p*-values of 0.837 and 0.726 for Stages IIA and IIB. For the post-pandemic period (*n* = 36), the distribution was Stage I in 2 patients (5.6%), Stage IIA in 5 (13.9%), Stage IIB in 3 (8.3%), Stage III in 16 (44.4%), Stage IVA in 1 (2.8%), and Stage IVB in 9 (25.0%). The *p*-values for Stages III, IVA, and IVB were 0.031, 0.002, and 0.043, respectively, indicating differences in the advanced stages across periods (Table 2), with stage IVB being significantly higher in the pandemic period, increasing by 16.1%.

Adenocarcinoma (tubular, papillary, mucinous, or mixed subtypes) was the most frequent histopathological finding, reported in 34 of 45 patients (75.6%) pre-pandemic, 28 of 40 (70.0%) during the pandemic, and 27 of 36 (75.0%) in the post-pandemic cohort (*p* = 0.659). Signet-ring cell (poorly cohesive) carcinoma comprised 6 of 45 cases (13.3%) in the pre-pandemic period, 7 of 40 (17.5%) during the pandemic, and 5 of 36 (13.9%) post-pandemic (*p* = 0.779). Lymphoma (diffuse large B-cell or follicular) was reported in 3 patients each (6.7% and 7.5%, respectively) in the pre-pandemic and pandemic groups, with a slight decrease to 2 out of 36 (5.6%) post-pandemic (*p* = 0.911). Undifferentiated carcinoma remained relatively rare across all three periods, detected in two patients (4.4%) pre-pandemic, two patients (5.0%) during the pandemic, and two patients (5.6%) in the post-pandemic cohort (*p* = 0.955). Overall, these findings suggest stable histopathological distributions despite the disruptions caused by the pandemic (Table 3).

For non-cardia gastric cancer (NCGC), the pre-pandemic group showed *H. pylori* positivity in 24 out of 33 patients (72.7%), during the pandemic, this was 20 out of 29 patients (69.0%), and post-pandemic, this was 19 out of 27 patients (70.4%), with an overall *p*-value of 0.915. In contrast, for cardia gastric cancer (CGC), *H. pylori* was positive in 5 out of 12 patients (41.7%) pre-pandemic, 4 out of 11 (36.4%) during the pandemic, and 5 out of 9 (55.6%) post-pandemic, with a *p*-value of 0.357. The overall comparison between NCGC and CGC across the time periods showed *H. pylori* prevalence rates of 72.7% vs. 41.7% in the pre-pandemic phase, 69.0% vs. 36.4% in the pandemic phase, and 70.4% vs. 55.6% in the post-pandemic phase (Table 4).

Overall, the proportion of patients presenting with upper GI bleeding differed significantly among the three periods (*p* = 0.046), peaking at 55.0% during the pandemic (versus 37.8% pre-pandemic and 44.4% post-pandemic). Among the bleeding cohort, antiplatelet or anticoagulant use was also highest in the pandemic group (59.1%, *p* = 0.029). Additionally, patients with bleeding during the pandemic had a significantly higher mean age (71.1 ± 9.5 years) compared to those pre- and post-pandemic (*p* = 0.045), as seen in Table 5.

**Figure 2:**
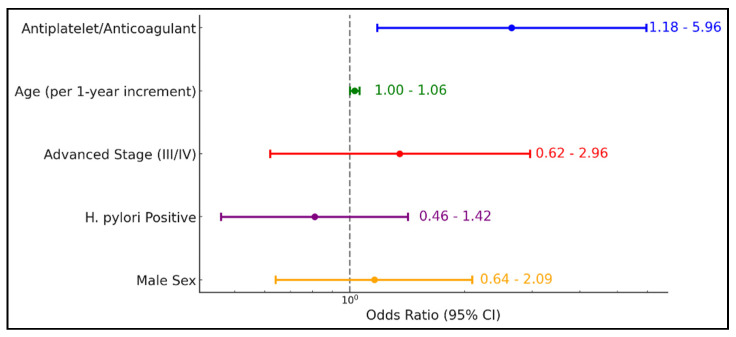
Logistic regression analysis for upper GI bleeding.

Table 6 and Figure 2 demonstrate that antiplatelet or anticoagulant therapy significantly increases the odds of upper GI bleeding by more than twofold (OR = 2.65, 95% CI 1.18–5.96; *p* = 0.018). While age exhibited a borderline effect (OR = 1.03, 95% CI 1.00–1.06; *p* = 0.065), advanced disease stage (III/IV), H. pylori positivity, and male sex did not show statistically significant associations with bleeding risk (Table 6).

Table 7 indicates that advanced tumor stage (III/IV) is the strongest predictor of one-year mortality (HR = 2.74, 95% CI 1.42–5.12; *p* = 0.002), followed by a diagnosis made during the COVID-19 pandemic (HR = 1.66, 95% CI 1.19–3.61; *p* = 0.010) and older age (≥70 years) (HR = 1.88, 95% CI 1.02–3.46; *p* = 0.043). Neither H. pylori positivity (HR = 0.82, 95% CI 0.47–1.42; *p* = 0.474) nor antiplatelet/anticoagulant use (HR = 1.26, 95% CI 0.73–2.17; *p* = 0.415) significantly affected mortality risk, and tumor location (cardia vs. non-cardia) also showed no significant impact (HR = 1.15, 95% CI 0.63–2.09; *p* = 0.642).

## 4. Discussion

### 4.1. Current Findings and Literature Findings

Our findings highlight the pandemic’s influence on gastric cancer care in Western Romania. Notably, a greater proportion of patients presented at Stage III and IVB post-pandemic, suggesting that diagnostic delays and reduced endoscopic capacity contributed to late detection [14,17,18,19]. Stage IVA, conversely, decreased in prevalence, although the overall burden of advanced disease remained high. Earlier studies in Japan and other regions corroborate shifts toward higher stages of GI cancers during COVID-19 [14,15,16].

*H. pylori* consistently showed a stronger association with NCGC, aligning with established research [4,6,8]. Although CGC infection rates trended upward post-pandemic (from 36.4% to 55.6%), statistical significance was not reached within that subgroup alone. The literature from East Asia supports the modest role of *H. pylori* in CGC [8], while studies from Europe and the USA historically report weaker correlations [25]. Further prospective research is warranted to verify whether changing patterns in Romania signal a broader epidemiological shift or sampling variation.

Upper GI bleeding was frequently encountered, especially among patients on antiplatelet/anticoagulant therapy, with 53.8% among those taking anticoagulants or antiplatelets compared to 32.9% in non-bleeders (OR = 2.65). This is clinically relevant for cardiovascular risk management, as concurrent GI bleeding may delay oncologic therapies and exacerbate morbidity [22,23,24]. Although advanced stage was not a statistically significant bleeding predictor in this study, it remains a clinically relevant concern given the frailty of these patients.

Survival analyses confirmed that advanced-stage disease and older age are the primary drivers of one-year mortality (HR = 2.74 and HR = 1.88, respectively). Neither *H. pylori* status nor tumor location independently influenced survival, emphasizing that early detection and prompt treatment remain paramount. These findings underscore the importance of maintaining diagnostic vigilance and sufficient endoscopy capacity even amid health crises to prevent escalation of disease severity and mortality. However, it is interesting that no significant differences in survival were noted, despite the reported delays. This suggests that the existing healthcare protocols may possess an inherent robustness, capable of maintaining patient outcomes even under strained conditions. This study raises questions about the scalability of these findings and whether larger datasets might reveal more nuanced effects. Moreover, it would be interesting to ascertain if similar resilience is observed across other centers in Romania, which could indicate the widespread efficacy of current medical practices during unprecedented times.

Although the exact role of *H. pylori* in CGC remains unclear, it is a well-established contributor to NCGC. Yang, L. et al. [8] conducted a study in 2021 in China that evaluated the connections between the presence of H. pylori and the possibility of developing NCGC and CGC among Chinese individuals. In NGCG, the antibody prevalence for H pylori reached 94.4%, while in CGC, this percentage was 92.2%. This research establishes a robust correlation between the presence of *H. pylori* and an elevated incidence of both NCGC and CG malignancies in the Chinese demographic [8]. A recent systematic review by Gu, J et al. [25], which included 27 papers, found that *H. pylori* is strongly associated with NCGC across both Asian and European/North American groups and that it is positively associated with CGC among Asian people but inversely associated with European/American people [25]. Similarly, in multiplex antibody assays, *H. Pylori*-specific antibodies were identified as a pathogenic factor for NCGC [26,27,28,29,30,31,32]. Our results corroborate previous research indicating a robust relationship between *H. pylori* and both kinds of gastric cancer, with the most significant correlation found in NCGC.

In 2022, during the pandemic, upper GI bleeding was negatively associated with *H. pylori* infection. *H. Pylori* infection was also linked with the COVID-19 pandemic era. It was lowest in the pre- and post-pandemic eras but was significant in the year 2022. Studies conducted by Balamtekin et al. and Zhang et al. suggested that people infected with *H. pylori* were more exposed to the risk of severe COVID-19 and SARS-CoV-2 infection [33,34].

Regarding survival rates, in a similar manner, the study conducted by Shigenobu et al. [35] reported a noticeable increase in mortality among patients diagnosed with gastric cancer during the COVID-19 pandemic. Using data from the Hiroshima Prefecture, the researchers found that the pandemic period (2020 and 2021) saw a significant increase in mortality, with a crude hazard ratio of 1.37 and an adjusted HR of 1.17, when compared to the pre-pandemic period (2018 and 2019). This study illustrated the substantial impact that the reduction in upper gastrointestinal endoscopies and subsequent delayed GC diagnoses had on patient outcomes, indicating a mortality rate of 374 events per 1000 person-years during the pandemic versus 278 events per 1000 person-years before the pandemic.

Conversely, a retrospective cohort study analyzing patients with high-risk gastrointestinal cancers during the COVID-19 pandemic, utilizing data from the National Cancer Database, portrayed a slightly different scenario [36]. This study encompassed 156,937 patients, finding that while there was a temporary decrease in new HRGI cancer diagnoses and a shift toward more advanced stages at diagnosis in early 2020, the 1-year mortality rates remained statistically unchanged between 2020 and the preceding two years. Specifically, the 1-year survival rate in 2020 was 47.4%, marginally lower than the 50.7% observed in 2018 and 2019, but this difference was not statistically significant (hazard ratio, 0.99; 95% CI, 0.97–1.01). This suggests that despite initial disruptions in cancer diagnosis and care, the overall survival outcomes did not deteriorate significantly.

Therefore, strengthening endoscopy services, reinforcing *H. pylori* eradication, and carefully balancing antithrombotic therapy are pivotal for improving GC outcomes, particularly during healthcare disruptions.

### 4.2. Study Limitations

This single-center study provides a comprehensive overview of GC patterns over three distinct pandemic-related periods, incorporating detailed histopathological data and rigorous staging. However, the retrospective design and modest sample size may limit generalizability. A limitation of this study is that, due to the institution’s continued reliance on a hybrid system of paper and electronic records, the exact percentage of missing variables remains unknown. Future research using multicenter data could validate these trends and help evaluate targeted interventions—such as telemedicine triage or prioritized endoscopy—to mitigate delays in GC diagnosis during healthcare system disruptions.

## 5. Conclusions

This study demonstrates a post-pandemic rise in advanced gastric cancer (particularly Stages III and IVB) in Western Romania, underscoring the negative impact of delayed diagnostic endoscopy. *H. pylori* continues to play a critical role in non-cardia GC, although an emerging increase in CGC infection warrants further investigation. Antiplatelet/anticoagulant use significantly raises the risk of upper GI bleeding, highlighting the need for careful management of antithrombotic agents in GC patients.

## Figures and Tables

**Table 1:** Baseline demographics and comorbidities.

Variables	Pre-Pandemic (*n* = 45)	Pandemic (*n* = 40)	Post-Pandemic (*n* = 36)	*p*-Value
Age (mean ± SD, years)	66.2 ± 9.7	68.5 ± 10.3	70.4 ± 11.2	0.149 (ANOVA)
Male Sex, *n* (%)	29 (64.4)	28 (70.0)	27 (75.0)	0.417 (χ^2^)
Hypertension, *n* (%)	28 (62.2)	22 (55.0)	25 (69.4)	0.381 (χ^2^)
Heart Failure, *n* (%)	19 (42.2)	16 (40.0)	16 (44.4)	0.922 (χ^2^)
Diabetes, *n* (%)	9 (20.0)	9 (22.5)	7 (19.4)	0.932 (χ^2^)
Anemia *, *n* (%)	37 (82.2)	34 (85.0)	29 (80.6)	0.835 (χ^2^)
BMI > 30 (Obesity), *n* (%)	15 (33.3)	13 (32.5)	11 (30.6)	0.935 (χ^2^)
Smoker, *n* (%)	17 (37.8)	18 (45.0)	14 (38.9)	0.692 (χ^2^)

* Anemia at admission defined as hemoglobin < 12 g/dL (female) or <13 g/dL (male).

**Table 2:** TNM staging distribution across three periods.

TNM Stage	Pre-Pandemic (*n* = 45)	Pandemic (*n* = 40)	Post-Pandemic (*n* = 36)	*p*-Value (χ^2^)
Stage I	5 (11.1)	3 (7.5)	2 (5.6)	0.679
Stage IIA	8 (17.8)	7 (17.5)	5 (13.9)	0.837
Stage IIB	6 (13.3)	5 (12.5)	3 (8.3)	0.726
Stage III	9 (20.0)	10 (25.0)	16 (44.4)	0.031
Stage IVA	13 (28.9)	5 (12.5)	1 (2.8)	0.002
Stage IVB	4 (8.9)	10 (25.0)	9 (25.0)	0.043

**Table 3:** Histopathological subtypes of gastric cancer.

Histopathology	Pre-Pandemic (*n* = 45)	Pandemic (*n* = 40)	Post-Pandemic (*n* = 36)	*p*-Value (χ^2^)
Adenocarcinoma (Tubular/Papillary/Mucinous/Mixed)	34 (75.6)	28 (70.0)	27 (75.0)	0.659
Signet Ring Cell (Poorly Cohesive)	6 (13.3)	7 (17.5)	5 (13.9)	0.779
Lymphoma (Diffuse Large B-cell, Follicular)	3 (6.7)	3 (7.5)	2 (5.6)	0.911
Undifferentiated Carcinoma	2 (4.4)	2 (5.0)	2 (5.6)	0.955

**Table 4:** *Helicobacter pylori* prevalence in cardia vs. non-cardia tumors.

Variables	Pre-Pandemic	Pandemic	Post-Pandemic	*p*-Value (χ^2^)
NCGC, *H. pylori*-Positive	24/33 (72.7%)	20/29 (69.0%)	19/27 (70.4%)	0.915
CGC, *H. pylori*-Positive	5/12 (41.7%)	4/11 (36.4%)	5/9 (55.6%)	0.357
Overall (NCGC vs. CGC)	72.7 vs. 41.7%	69.0 vs. 36.4%	70.4 vs. 55.6%	0.041

**Table 5:** Upper GI bleeding distribution by study period.

Variable	Pre-Pandemic (*n* = 45)	Pandemic (*n* = 40)	Post-Pandemic (*n* = 36)	*p*-Value (χ^2^ or *t*-Test)
No Bleeding, *n* (%)	28 (62.2)	18 (45.0)	20 (55.6)	0.046 (χ^2^)
Bleeding, *n* (%)	17 (37.8)	22 (55.0)	16 (44.4)	0.046 (χ^2^)
Antiplatelet/Anticoagulant Use (among Bleeding Patients), *n* (%)	7 (41.2)	13 (59.1)	7 (43.8)	0.029 (χ^2^)
Mean Age ± SD (years) (Bleeding Group)	66.3 ± 10.2	71.1 ± 9.5	69.8 ± 8.9	0.045 (ANOVA)
Advanced Stage (III/IV) (among Bleeding Patients), *n* (%)	11 (64.7)	16 (72.7)	10 (62.5)	0.551 (χ^2^)
H. pylori Positivity (among Bleeding Patients), *n* (%)	8 (47.1)	10 (45.5)	8 (50.0)	0.938 (χ^2^)

**Table 6:** Logistic regression analysis for upper GI bleeding.

Risk Factor	OR (95% CI)	*p*-Value
Antiplatelet/Anticoagulant	2.65 (1.18–5.96)	0.018
Age (per 1-year increment)	1.03 (1.00–1.06)	0.065
Advanced Stage (III/IV)	1.35 (0.62–2.96)	0.448
*H. pylori* Positivity	0.81 (0.46–1.42)	0.468
COVID-19 period	1.93 (1.09–4.27)	0.006
Male Sex	1.16 (0.64–2.09)	0.631

**Table 7:** One-year mortality risk in gastric cancer: Cox regression.

Predictor	HR (95% CI)	*p*-Value
Advanced Stage (III/IV)	2.74 (1.42–5.12)	0.002
Diagnosis during COVID-19 pandemic	1.66 (1.19–3.61)	0.010
Age ≥ 70 years	1.88 (1.02–3.46)	0.043
*H. pylori* Positivity	0.82 (0.47–1.42)	0.474
Antiplatelet/Anticoagulant	1.26 (0.73–2.17)	0.415
Cardia vs. Non-Cardia	1.15 (0.63–2.09)	0.642

## Data Availability

The original contributions presented in the study are included in the article. Further inquiries can be directed to the corresponding author.
